# Gandouling ameliorates liver injury in Wilson's disease through the inhibition of ferroptosis by regulating the HSF1/HSPB1 pathway

**DOI:** 10.1111/jcmm.70018

**Published:** 2024-09-02

**Authors:** Chenling Zhao, Jie Chen, Liwei Tian, Yuya Wen, Mingcai Wu, Lulu Tang, An Zhou, Wenting Xie, Ting Dong

**Affiliations:** ^1^ Department of Neurology The First Affiliated Hospital of Anhui University of Chinese Medicine Hefei China; ^2^ School of Basic Medical Sciences Wannan Medical College Wuhu China; ^3^ Key Laboratory of Xin'An Medicine Ministry of Education Hefei China; ^4^ The Experimental Research Center Anhui University of Chinese Medicine Hefei China

**Keywords:** ferroptosis, Gandouling, HSF1/HSPB1 pathway, liver injury, Wilson's disease

## Abstract

Ferroptosis, an iron‐dependent form of cell death, plays a crucial role in the progression of liver injury in Wilson's disease (WD). Gandouling (GDL) has emerged as a potential therapeutic agent for preventing and treating liver injury in WD. However, the precise mechanisms by which GDL mitigates ferroptosis in WD liver injury remain unclear. In this study, we discovered that treating Toxic Milk (TX) mice with GDL effectively decreased liver copper content, corrected iron homeostasis imbalances, and lowered lipid peroxidation levels, thereby preventing ferroptosis and improving liver injury. Bioinformatics analysis and machine learning algorithms identified *Hspb1* as a pivotal regulator of ferroptosis. GDL treatment significantly upregulated the expression of HSPB1 and its upstream regulatory factor HSF1, thereby activating the HSF1/HSPB1 pathway. Importantly, inhibition of this pathway by NXP800 reversed the protective effects of GDL on ferroptosis in the liver of TX mice. In conclusion, GDL shows promise in alleviating liver injury in WD by inhibiting ferroptosis through modulation of the HSF1/HSPB1 pathway, suggesting its potential as a novel therapeutic agent for treating liver ferroptosis in WD.

## 1 INTRODUCTION

Wilson's disease (WD) is an autosomal‐recessive disorder of copper metabolism. The global prevalence rates of WD range from approximately 1/10,000 to 1/30,000, with a significantly higher proportion of WD patients in China compared to Western countries.^1^ Its aetiology is highly related to a genetic defect in the ATPase copper transporting beta gene (ATP7B).^2^ The ATP7B gene is primarily expressed in the liver and is responsible for copper transport within hepatocytes; therefore, the liver is the initial defective organ and the most affected by copper accumulation.^3^, ^4^, ^5^ For most WD patients, the first clinical symptom is liver injury, the severity of which is closely related to prognosis.^6^ Liver injury occurs throughout the entire disease course, starting from birth, with pathological manifestations ranging from asymptomatic subtle morphological changes to acute/chronic hepatitis, cirrhosis and liver failure.^2^, ^7^ Therefore, clarifying the mechanism of liver injury in WD is of significant importance for its treatment and prognosis.

Ferroptosis is a novel type of programmed cell death driven by the accumulation of iron‐dependent lipid peroxides.^8^ The unique characteristics of ferroptosis distinguish it from other types of cell death programmes, including apoptosis, pyroptosis and autophagy.^9^ In WD patients, excessive accumulation of copper can lead to impaired ceruloplasmin (CP, which functions as a copper transporter and has ferrous oxidase activity^10^) synthesis and inability to complete the ferrous iron oxidation process, which causes a reduction in the amount of circulating iron and an increase in iron deposits.^11^ Further research has found that copper overload is accompanied by iron overload, and the excessive accumulation of iron is the direct cause of ferroptosis.^12^ In recent years, ferroptosis as a key contributor to WD liver injury has been intensely studied. Sun et al. observed the characteristic manifestations of ferroptosis in the livers of WD model mice. Furthermore, in vitro experiments have revealed that high cellular copper loads can trigger ferroptosis.^5^ Regulation of ferroptosis can delay the pathological progression of liver injury in WD.

Gandouling (GDL), with approval number Z20050071, is an effective Chinese herbal compound preparation developed by the First Affiliated Hospital of Anhui University of Chinese Medicine, which possesses independent intellectual property rights. It is known for its therapeutic effects in heat clearance, detoxification, blood stasis removal, knot dispersion, gallbladder function promotion, as well as copper discharge.^13^ GDL is primarily composed of Coptis chinensis Franch, Salvia miltiorrhiza Bunge, Spatholobus suberectus Dunn, Curcuma aeruginosa Roxb, Rheum palmatum L, and Curcuma longa L in ratios of 27:14:14:12:12:12 (the plant names were checked on 28 December 2023, http://www.theplantlist.org).^14^ A systematic analysis and identification of the chemical components of GDL have been carried out by our research group, which has laid the foundation for quality control and the study of pharmacological substances of GDL.^15^ In accordance with the 2023 ‘Guidelines for Diagnosis and Treatment of WD with Integrated Traditional and Western Medicine’ in China, GDL has been recommended for the improvement of TCM syndrome scores and levels of serological markers of liver function in patients with WD‐mediated liver injury. Previous research has demonstrated that the predominant chemical component of GDL, curcumin can exert a hepatoprotective effect on WD by promoting copper excretion and inhibiting ferroptosis.^5^ However, whether GDL protects against liver injury in WD by inhibiting ferroptosis and its specific regulatory mechanism remain unclear.

Hence, in the present study, we sought to explore whether the liver injury in WD was associated with ferroptosis and to reveal the specific mechanism using bioinformatics analysis, machine learning algorithms, and in vivo experiments. We speculated that GDL ameliorates liver injury in WD through the inhibition of ferroptosis by regulating the HSF1/HSPB1 pathway. The findings of our study contribute to a better understanding of the molecular‐level regulatory mechanisms underlying liver injury in WD, and provide evidence supporting GDL as a promising candidate drug for the treatment of liver ferroptosis in WD.

## 2 MATERIALS AND METHODS

### 2.1 Animals and groups

The Toxic Milk (TX) mice were initially identified in 1987 within the C3H/HeJ animal resource population at Jackson Laboratory in Bar Harbour, Maine. Similar to *Atp7b* ^−/−^ mice, TX mice exhibit significant hepatologic phenotypes characterized by progressive liver injury and copper accumulation due to mutations in the *Atp7b* gene. Specifically, TX mice carry a spontaneous recessive point mutation at position 2135 in exon 8 of the *Atp7b* gene, resulting in a missense mutation (G712D). This mutation closely resembles those found in human cases of WD. With an 82% sequence homology to human *ATP7B*, the TX mice are widely recognized as the most representative animal model for studying WD, mirroring comparable clinical characteristics and disease progression.^16^, ^17^

For experimental purposes, the TX mice (strain: C3HeB/FeJAtp7btx‐J/J) and homologous wild‐type control mice (aged 6 months, weighing 20–25 g) were purchased from the Jackson Laboratory (Bar Harbour, ME, USA) and bred in the specific pathogen free (SPF) class animal laboratory of Anhui Agricultural University (Anhui, China). They were housed in a controlled environment (temperature: 25 ± 2°C, humidity: 40%–60%, illumination: 12 h light–dark cycle) and provided with an adequate food and water.

After normal feeding for 1 week, the male mice were selected for subsequent research, and they were equally randomized into four groups (*n* = 8 per group): (1) control group, (2) model group, (3) GDL group, and (4) Ferrostatin‐1 (Fer‐1, HY‐100579, MCE, China) group. According to the equivalent dosage conversion for WD patients and experimental mice, the optimum dosage administered to the GDL group via oral gavage was 1.16 g/kg per day.^15^, ^18^ The stomachs of mice in the control and model groups were irrigated with equal volumes of normal saline, for a total of six successive weeks. The dosage, time and administration pathway of Fer‐1 group were based on previous study (0.001 g/kg/day, i.p., once daily for 14 days).^19^

To explore whether GDL ameliorates liver injury in WD through the inhibition of ferroptosis by regulating the HSF1/HSPB1 pathway, another batch of mice was randomly divided into four groups (*n* = 8) after a week of acclimatization: (1) control group, (2) model group, (3) GDL group and (4) GDL + NXP800 (HY‐145927, MCE, China) group. The dosage, time, and administration pathway of GDL + NXP800 group were based on the product description and preliminary experiment result (0.001 g/kg, i.g., once daily for 7 days). The remaining groups were treated in the same way as described above.

**FIGURE 1:**
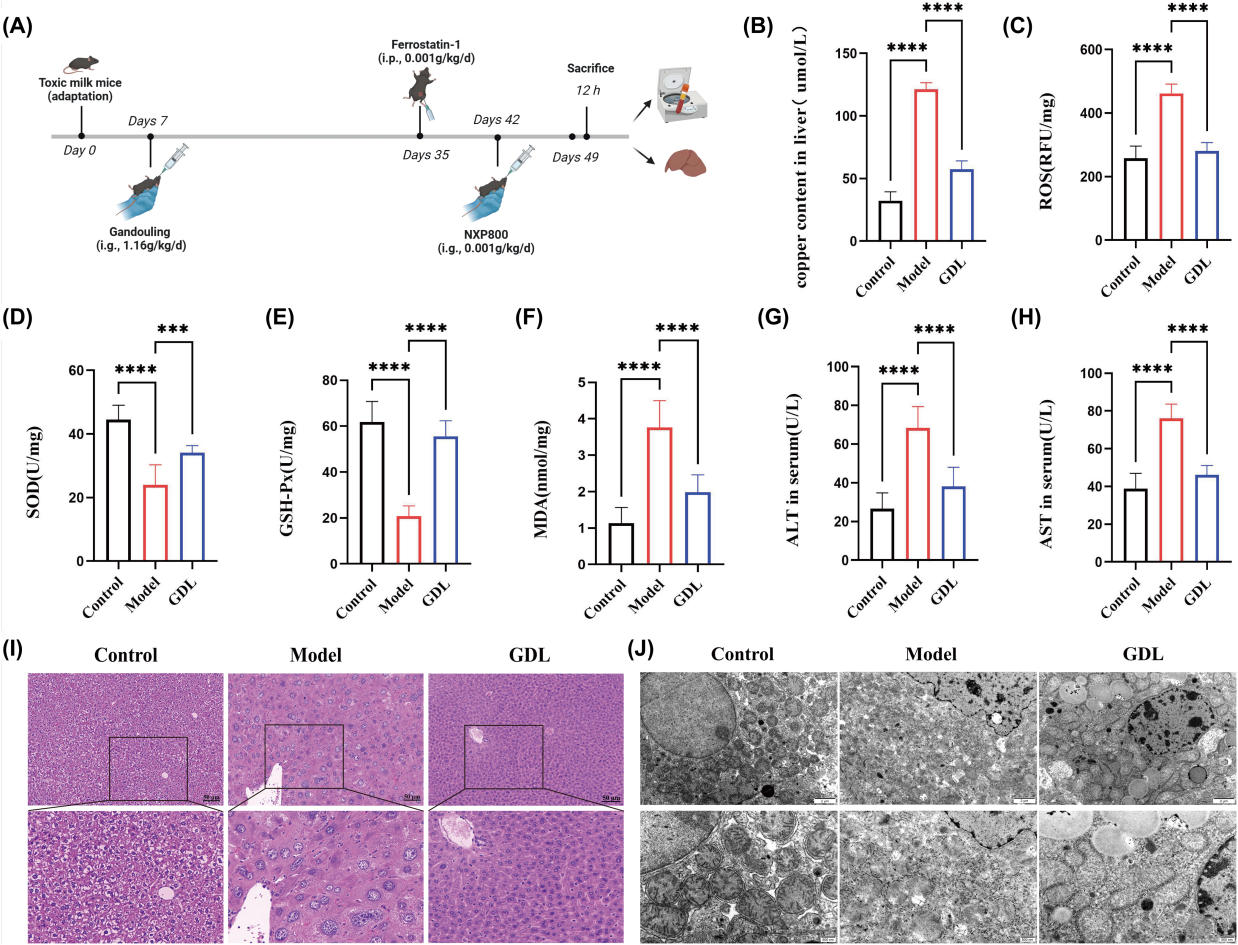
GDL relieves liver injury in TX mice. (A) Schematic of the experimental protocol. (B) Copper content in the liver (*n* = 8). (C–F) Levels of oxidative stress indicators ROS, SOD, GSH‐Px, and MDA in the liver (*n* = 8). (G–H) Serum ALT and AST levels (*n* = 8). (I) H&E staining (scale bar = 50 μm). (J) Transmission electron microscopy images (scale bar = 2 μm and 500 nm). Data are presented as mean ± SD. ***p < 0.001, ****p < 0.0001.

After the last administration, a fasting period of 12 h was required. Sodium pentobarbital (50 mg/kg, i.p.) was used to anesthetize all the mice. Venous blood was then collected from the retroorbital sinus, centrifuged at 4000 rpm for 10 min, and the supernatant was stored at −80°C. Liver tissue was excised and rinsed with normal saline. The experimental flow chart is detailed in Figure 1A.

All protocols regarding animal care and experiments were reviewed and approved by the Animal Ethics Review Committee of Anhui Agricultural University (AHAU2022021).

### 2.2 Identification of ferroptosis core genes by bioinformatics analysis and machine learning algorithms

The raw dataset GSE125637^20^ including gene expression data for wild type and WD model mice liver tissues was downloaded from the Gene Expression Omnibus (GEO, https://www.ncbi.nlm.nih.gov/geo/) database.^21^ Ferroptosis‐related genes (FRGs), including driver, suppressor and marker genes, were retrieved from the FerrDb (http://www.zhounan.org/ferrdb) database.^22^ Based on the limma package in R software, the dataset was analysed to obtain differentially expressed genes (DEGs) between WD and wild type mice liver tissues (When multiple probes identified the same gene, the average value was calculated to determine its expression).^23^ |log2 Fold change (FC)| >1 and *p* < 0.05 were set as the criteria for identifying DEGs. Subsequently, the crossover genes between DEGs and FRGs were identified as differentially expressed ferroptosis‐related genes (DE‐FRGs). The clusterProfiler package was used to identify the functional enrichment characteristics of DE‐FRGs,^24^ and to explore their potential biological processes (BP), cellular components (CC), molecular functions (MF), and important signalling pathways.^25^, ^26^ The screening condition was limited to *p* < 0.05. Two machine learning algorithms, including least absolute shrinkage and selection operator (LASSO), and support vector machine‐recursive feature elimination (SVM‐RFE) were adopted to further filter DE‐FRGs for WD. LASSO is a regression method for variable selection to improve the predictive accuracy and comprehensibility of statistical models.^27^ SVM‐RFE can remove redundant factors and retains only variables related to the outcome, which helps to improve the diagnostic performance of the prediction model.^28^ The glmnet and e1071 packages were used to perform LASSO regression and SVM‐RFE analysis.^29^, ^30^, ^31^ The intersection genes of LASSO and SVM‐RFE were considered as core genes of DE‐FRGs in WD.

### 2.3 Histological assessment

Fresh liver tissues were fixed in 4% paraformaldehyde for more than 24 h, dehydrated with ethanol, and embedded in paraffin. Sections 5 μm thick were prepared for haematoxylin–eosin (H&E) staining. Histopathological changes in these sections were observed under a microscope (CX41, OLYMPUS, Japan).

### 2.4 Ultrastructural changes

Fresh liver tissues were fixed in 2.5% glutaraldehyde fixative, stored at 4°C for 2 h, subjected to dehydration, osmotic embedding and finally sectioned into ultrathin sections (70 nm). The ultrathin sections were observed for ultrastructure using transmission electron microscopy (JEM1400, JEOL, Japan).

### 2.5 Immunohistochemical staining

Liver tissue sections (2 μm) were deparaffinized in xylene through three 15‐min cycles, followed by a gradient dehydration process utilizing varying ethanol concentrations, with each step lasting 5 min. The sections were then rinsed with distilled water until they appeared clear and transparent. Antigen retrieval was conducted, and endogenous peroxidase activity was inhibited. The sections were incubated with primary antibodies directed against HSF1 (4356S, Cell Signaling, USA), HSPB1 (50353S, Cell Signaling, USA) and 4‐Hydroxynonenal (4‐HNE, HY‐P81208, MCE, China) at 37°C for 60 min. Subsequently, they were labelled with HRP‐conjugated secondary antibodies and incubated again at 37°C for 20 min. Horseradish peroxidase‐labelled streptavidin solution was added and allowed to incubate for 30 min at the same temperature. The reaction was visualized using DAB substrate, followed by counterstaining with haematoxylin. Finally, the sections were mounted and examined under a CX41 microscope (OLYMPUS, Japan).

### 2.6 Prussian blue staining

To determine the iron content, liver sections were stained using a Prussian blue iron stain kit (B034, Solarbio, China). The paraffin‐embedded sections were dewaxed and subsequently washed in distilled water for 1 min, stained with Prussian blue staining solution for 30 min, thoroughly rinsed with distilled water for 5 min, and lightly stained with nuclear red solution for 10 min, followed by another rinse with water. Finally, the sections were dehydrated, cleared and mounted in a neutral mounting medium. Positive staining was indicated by blue coloration.

### 2.7 Measurement of copper and ferrous iron content

The copper content in liver tissues was determined through colorimetry, following the protocol specified in the copper assay kit (E010‐1‐1, Nanjing Jiancheng Bioengineering Institute, China). Similarly, the content of ferrous iron (Fe^2+^) in liver tissues was quantified using an iron assay kit (FI‐W96‐N 1620, Shanghai Enzyme‐linked Biotechnology Co., Ltd., China), according to the manufacturer's instructions.

### 2.8 Enzyme‐linked immunosorbent assay (ELISA)

The levels of alanine aminotransferase (ALT) and aspartate aminotransferase (AST) were detected in serum samples, while the levels of malondialdehyde (MDA), superoxide dismutase (SOD), glutathione peroxidase (GSH‐Px), and reactive oxygen species (ROS) were measured in liver tissues, using corresponding ELISA kits (C009‐2‐1, C010‐2‐1, A003‐1‐2, A001‐3‐2, A005‐1‐2, E004‐1‐1, Nanjing Jiancheng Bioengineering Institute, China). All assays were performed strictly according to the manufacturer's protocols to ensure the consistency and accuracy of the results.

### 2.9 Quantitative real‐time PCR (RT‐qPCR)

Total RNA was extracted from liver tissues using Trizol reagent (RN001‐50Rxns, ESscience, China). The reverse transcription kit (RT001, ESscience, China) was used to synthesize cDNA according to the provided instructions, and the resulting product was stored at −20°C. Quantitative PCR amplification was carried out using the SYBR kit (Q204‐01, EnzyArtisan, China) as per the manufacturer's guidelines. The analysis of gene expression was performed using the 2^−△△Ct^ method, with β‐actin serving as the internal control. The primer sequences were listed in Table 1.

**TABLE 1:** The primer sequences used for real‐time PCR assay.

Gene	Forward primer (5′–3′)	Reward primer (5′–3′)
Fth1	CTGGAACTGCACAAACTGGC	CTCTCATCACCGTGTCCCAG
Hspb1	ATGAGTGGTCGCAGTGGTTC	TTCGTGCTTGCCAGTGATCT
Hsf1	GCCCCTCTTCCTTTCTGCAT	TCATGTCGGGCATGGTCAC
Tfr1	TGAGTGGCTACCTGGGCTAT	CTCCTCCGTTTCAGCCAGTT
Gpx4	CCCGATATGCTGAGTGTGGTTTAC	TTTCTTGATTACTTCCTGGCTCCTG
β‐Actin	GAACCCTAAGGCCAACCGTG	CGACCAGAGGCATACAGGGA

### 2.10 Western blot (WB)

Protein lysate was used for total protein extraction from liver tissue. The protein concentration was determined using the BCA Protein Analysis Kit (P0010S, Beyotime, China). The extracted proteins were separated after 90 min of electrophoresis on 10% sodium dodecyl sulphate‐polyacrylamide gel electrophoresis (SDS‐PAGE) gels. Subsequently, the proteins were transferred to polyvinylidene fluoride (PVDF) membranes using a wet transfer method. The membranes were blocked with 5% milk at room temperature for 90 min and then incubated overnight at 4°C with the corresponding primary antibodies listed in Table 2. After the overnight incubation, the membranes were washed three times. HRP‐labelled goat anti‐rabbit/mouse IgG (H + L) antibody (BA1054/BA1050, Boster, China) was added and incubated at room temperature for 2 h. Finally, the membranes were treated with an ultra‐sensitive ECL solution (D046‐1, Bridgen, China). The protein intensity was analysed using ImageJ software (version 1.8.0), with β‐actin as the reference for normalizing protein expression of heat shock factor 1 (HSF1), heat shock protein beta‐1 (HSPB1), ferritin heavy chain 1 (FTH1), transferrin receptor 1 (TFR1), and glutathione peroxidase 4 (GPX4).

**TABLE 2:** The information of antibodies used in the present experiment.

Antibody	Species	Dilution ratio	Company (catalogue number)
HSPB1	Rabbit	1:1000	Cell Signaling, USA (50353S)
HSF1	Rabbit	1:1000	Cell Signaling, USA (4356S)
FTH1	Rabbit	1:1000	Proteintech, USA (11682‐1‐AP)
TFR1	Mouse	1:5000	Abcam, UK (ab269513)
GPX4	Mouse	1:1000	Proteintech, China (67763‐1‐lg)
β‐Actin	Rabbit	1:3000	Affinity, USA (AF7018)

### 2.11 Statistical analysis

The mean ± standard deviation (SD) was used to express quantitative data. Multiple groups were compared using one‐way analysis of variance, while two groups were compared using two independent sample *t*‐tests. Statistical significance was denoted by *p < 0.05. For more significant differences, **p < 0.01, ***p < 0.001 and ^****^ *P* < 0.0001 were indicated. Graphs were generated using GraphPad Prism (version 9.4.0) or R software (version 4.3.0).

## 3 RESULTS

### 3.1 GDL relieves liver injury in TX mice

To explore the underlying function of GDL on liver injury in TX mice, we initially examined the levels of liver copper content, serum liver function and liver oxidative stress indicators (Figure 1B–H). Compared to the control group, the model group exhibited significantly higher levels of copper content, ALT, AST, MDA and ROS, as well as a lower concentration of GSH‐Px and SOD. However, these changes were reversed by GDL. Furthermore, liver histological evaluation was performed using H&E staining. As illustrated in Figure 1I, no significant pathological abnormalities were observed in the liver tissue of the control group. In contrast, the model group displayed several histopathological changes, including liver structure disruption, irregular arrangement, increased hepatocyte necrosis and vacuole formation. Treatment with GDL effectively attenuated the degree of liver injury with better retention of native liver architecture. Regarding mitochondrial morphology, as shown by transmission electron microscopy (Figure 1J), the control group exhibited regular shape, normal structure, and clear mitochondrial cristae. However, liver mitochondria in the model group suffered significant injury, characterized by rupture of the outer mitochondrial membrane and loss of mitochondrial cristae. Treatment with GDL resulted in substantial reduction in mitochondrial injury compared to the model group. Collectively, these findings demonstrate that GDL exerted a protective effect against liver injury in TX mice.

### 3.2 Involvement of ferroptosis in liver injury in TX mice

**FIGURE 2:**
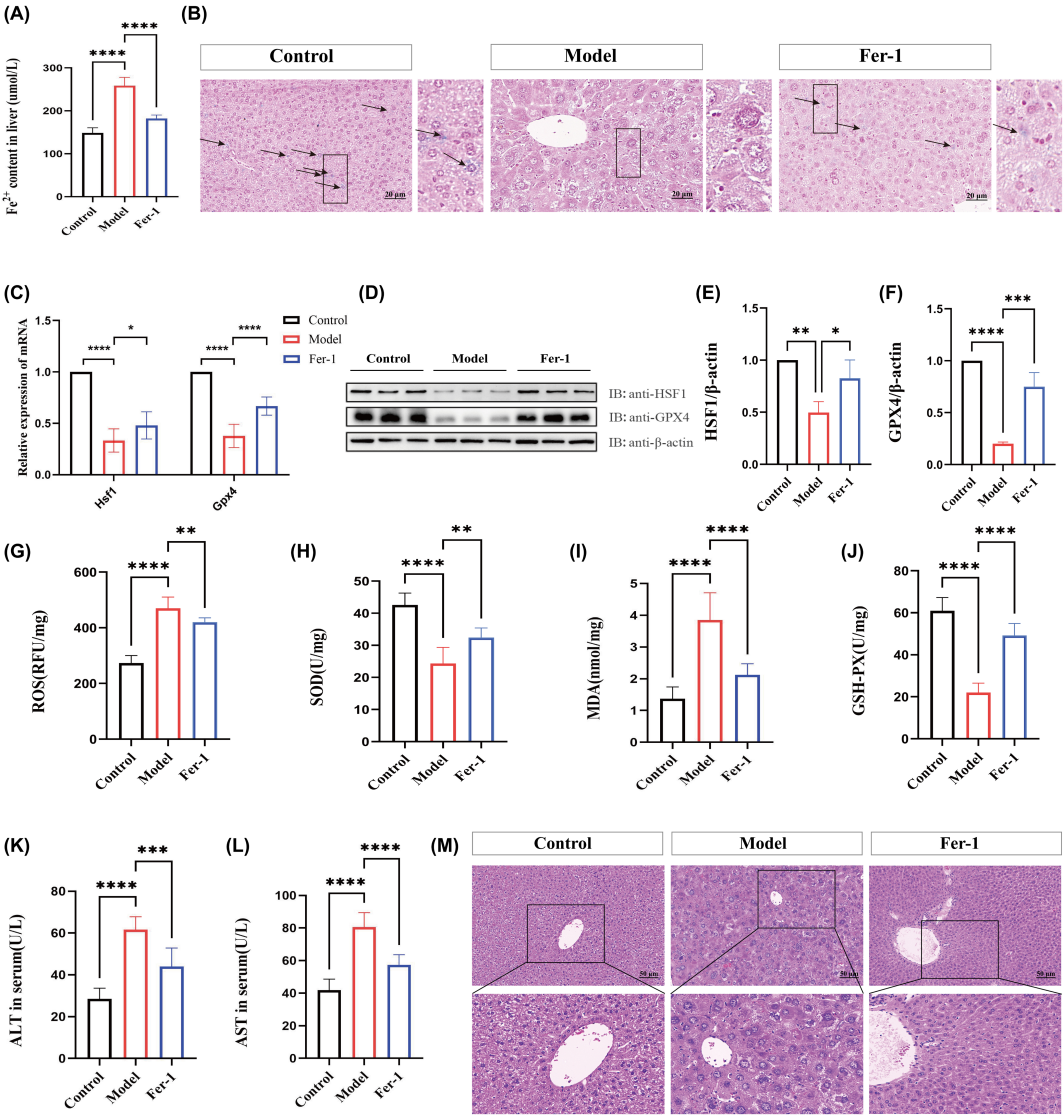
Involvement of ferroptosis in liver injury in TX mice. (A) Fe^2+^ content in the liver. (B) Prussian blue staining (scale bar = 20 μm). (C) mRNA expression levels of *Hsf1* and *Gpx4* in the liver (*n* = 9). (D–F) Protein expression levels and quantitative analysis of HSF1 and GPX4 in the liver (*n* = 3). (G–J) Levels of oxidative stress indicators ROS, SOD, MDA and GSH‐Px in the liver (*n* = 8). (K–L) Serum ALT and AST levels (*n* = 8). (M) H&E staining (scale bar = 50 μm). Data are presented as mean ± SD. *p < 0.05, **p < 0.01, ***p < 0.001, ****p < 0.0001.

To assess the role of ferroptosis in liver injury in TX mice, we administered Fer‐1, a recognized inhibitor of ferroptosis. The results indicated that Fer‐1 significantly decreased Fe^2+^ content in liver tissue (Figure 2A). Additionally, ferric iron (Fe^3+^) levels were quantified using a Prussian blue iron stain kit. It was observed that Fe^3+^ levels were reduced in the model group; however, intervention with Fer‐1 reversed this trend (Figure 2B). We further analysed two critical ferroptosis‐related markers, GPX4 and HSF1, in the liver. As shown in Figure 2C–F, both GPX4 and HSF1 exhibited downregulation at both mRNA and protein levels in the model group compared to the control group. Intervention with Fer‐1 markedly attenuated these markers in the liver. Additionally, Fer‐1 reduced levels of ALT, AST, MDA and ROS, while increasing concentrations of SOD and GSH‐Px in the model group, demonstrating effects similar to those observed with GDL treatment (Figure 2G–L). H&E staining indicated a significant reduction in liver histopathological injury in the Fer‐1 group, as depicted in Figure 2M. The above evidence suggests that ferroptosis plays a significant role in liver injury in TX mice.

### 3.3 Hspb1 is the core gene regulating ferroptosis

**FIGURE 3:**
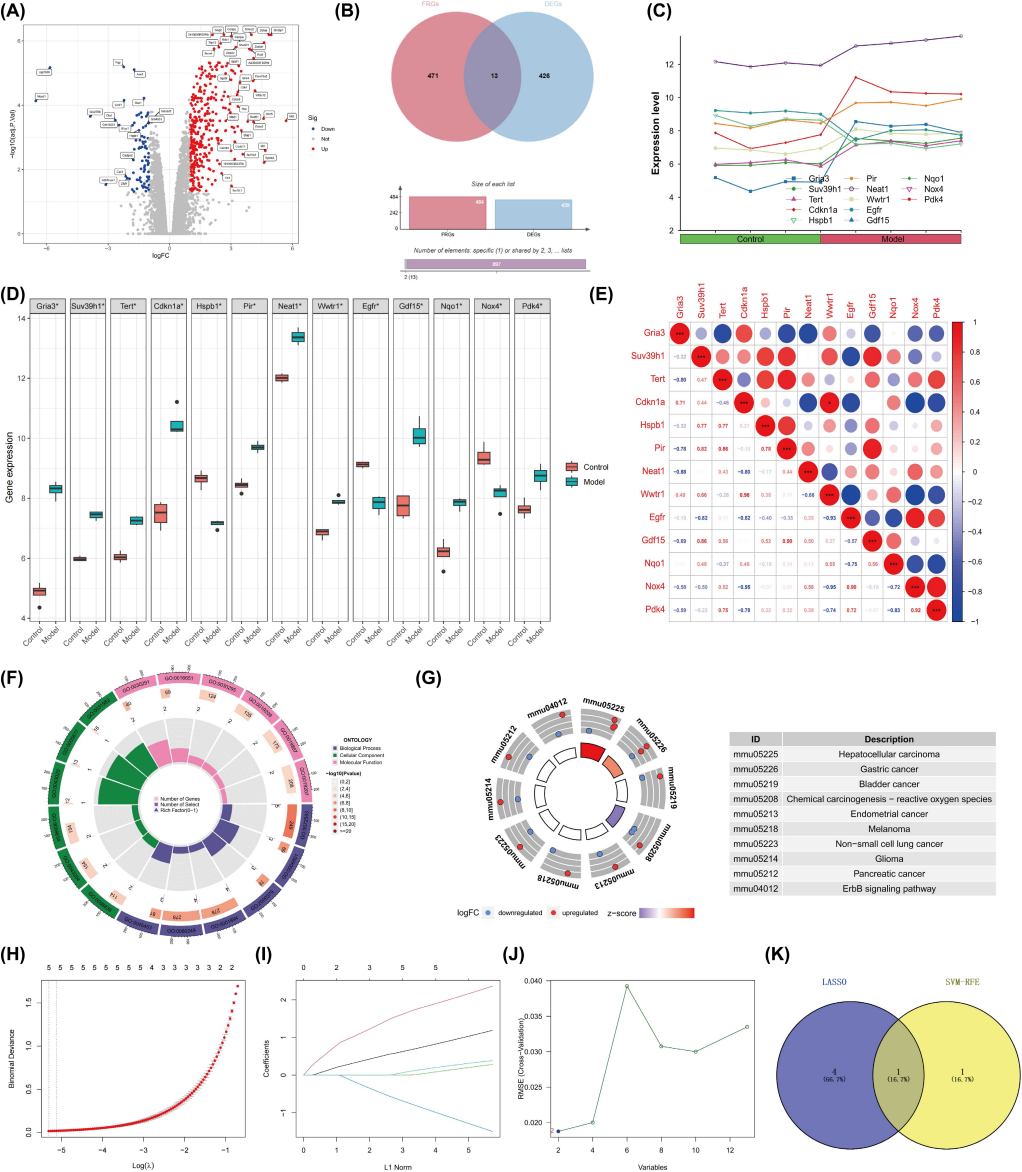
Identification of ferroptosis core genes in the WD mice model. (A) Volcano plot of the differentially expressed genes (DEGs). (B–E) Venn diagram, line plot, box plot, and correlation plot of the differentially expressed ferroptosis‐related genes (DE‐FRGs). (F–G) GO and KEGG enrichment analysis for DE‐FRGs. (H, I) Identification of five candidate DE‐FRGs using LASSO regression with 10‐fold cross‐validation. (J) Establishment of characteristic DE‐FRGs (*n* = 2) identified by SVM‐RFE. (K) Venn diagram of the core genes.

A total of 439 genes (including 357 up‐regulated genes and 82 down‐regulated genes) were identified as DEGs (Figure 3A). Four hundred and eighty‐four FRGs were retrieved from the FerrDb database. Through the crossover analysis of DEGs and FRGs, 13 genes were identified as DE‐FRGs, which can be visually represented in the Venn diagram, line plot and box plot (Figure 3B–D). Subsequently, correlation analysis was conducted, revealing that *Wwtr1* and *Cdkn1a* had higher positive correlation coefficients among the DE‐FRGs (*r* = 0.96), whereas *Nox4* and *Cdkn1a* (*r* = −0.95), as well as Nox4 and Wwtr1 (*r* = −0.95), exhibited larger negative correlation coefficients (Figure 3E). Gene Ontology (GO) analysis elucidated that DE‐FRGs were mainly enriched in BP terms, including ROS metabolic process, tissue homeostasis and response to nutrient levels. Regarding CC terms, the DE‐FRGs were mainly located in the perikaryon and postsynaptic density/specialization membrane. MF analysis showed that protein kinase inhibitor/activator activity, and oxidoreductase activity were the most significant items among DE‐FRGs (Figure 3F). Furthermore, Kyoto Encyclopedia of Genes and Genomes (KEGG) analysis illustrated that DE‐FRGs were mainly enriched in cancer‐related signalling pathways, ErbB signalling pathways, and chemical carcinogenesis‐reactive oxygen species (Figure 3G). By integrating the outcomes of two machine learning algorithms, LASSO and SVM‐RFE, the crucial DE‐FRGs for the WD process were determined. Five significant DE‐FRGs were identified through LASSO regression (Figure 3H,I). Additionally, SVM‐RFE screened two genes with the highest accuracy and lowest error (Figure 3J). The Venn diagram visualization demonstrated the intersection of DE‐FRGs from LASSO and SVM‐RFE (Figure 3K), leading to the identification of one core gene, *Hspb1*.

### 3.4 GDL inhibits ferroptosis by ameliorating iron homeostasis dysregulation in the liver of TX mice

**FIGURE 4:**
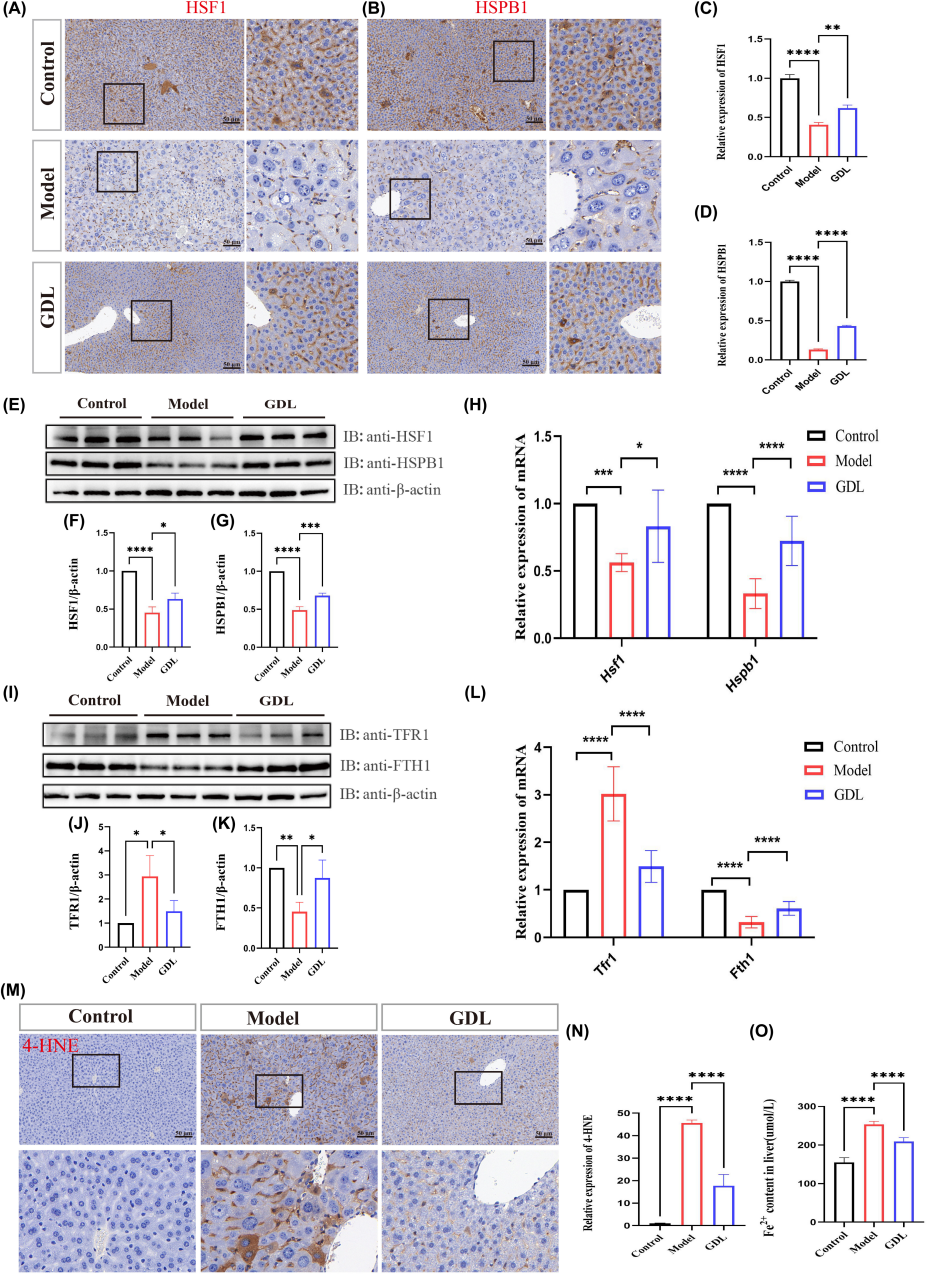
GDL inhibits ferroptosis by ameliorating iron homeostasis dysregulation in the liver of TX mice. (A–D) Immunohistochemical staining and quantitative analysis of HSF1 and HSPB1 in the liver (scale bar = 50 μm). (E–G) Protein expression levels and quantitative analysis of HSF1, and HSPB1 in the liver (*n* = 3). (H) mRNA expression levels of *Hsf1* and *Hspb1* in the liver (*n* = 9). (I–K) Protein expression levels and quantitative analysis of TFR1 and FTH1 in the liver (*n* = 3). (L) mRNA expression levels of *Tfr1* and *Fth1* in the liver (*n* = 9). (M, N) Immunohistochemical staining and quantitative analysis of 4‐HNE in the liver (scale bar = 50 μm). (O) Fe^2+^ content in the liver. Data are presented as the mean ± SD. *p < 0.05, **p < 0.01, ***p < 0.001, ****p < 0.0001.

To investigate the inhibitory effects of GDL on liver ferroptosis in TX mice, we evaluated iron homeostasis in the liver. Immunohistochemical staining detected the expression of HSPB1 and its upstream regulator, HSF1. Compared to the control group, the model group exhibited significantly reduced expression of HSPB1 and HSF1 in the liver. However, GDL effectively reversed these alterations (Figure 4A–D). The protein and mRNA levels of HSPB1 and HSF1 also showed trends consistent with the aforementioned results (Figure 4E–H). Additionally, GDL reversed the increased protein and mRNA levels of TFR1 in the liver of TX mice compared to those in the model group, and restored the levels of FTH1 (Figure 4I–L). Moreover, elevated levels of 4‐HNE (a marker of lipid peroxidation and ferroptosis) and Fe^2+^ were observed in the model group. However, GDL effectively reduced the levels of both 4‐HNE and Fe^2+^ (Figure 4M–O). These findings indicate that GDL can inhibit ferroptosis by ameliorating dysregulation of iron homeostasis in the liver of TX mice.

### 3.5 GDL ameliorates liver injury in TX mice through the inhibition of ferroptosis by regulating the HSF1/HSPB1 pathway

**FIGURE 5:**
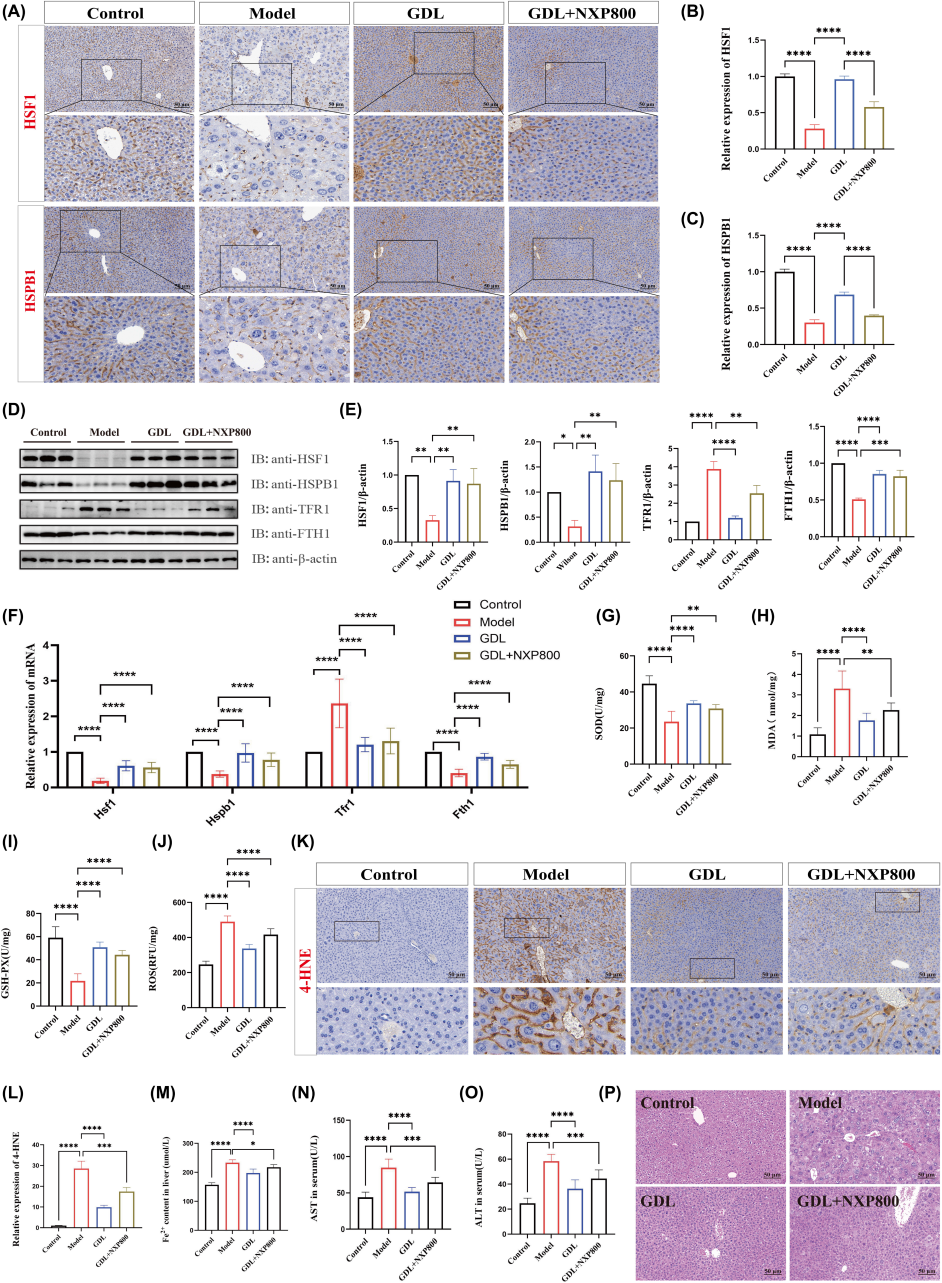
GDL ameliorates liver injury in TX mice through the inhibition of ferroptosis by regulating the HSF1/HSPB1 pathway. (A–C) Immunohistochemical staining and quantitative analysis of HSF1 and HSPB1 in the liver (scale bar = 50 μm). (D, E) Protein expression levels and quantitative analysis of HSF1, HSPB1, TFR1 and FTH1 in the liver (*n* = 3). (F) mRNA expression levels of *Hsf1*, *Hspb1*, *Fth1* and *Tfr1* in the liver (*n* = 9). (G–J) Levels of oxidative stress indicators SOD, MDA, GSH‐Px and ROS in the liver (*n* = 8). (K, L) Immunohistochemical staining and quantitative analysis of 4‐HNE in the liver (scale bar =50 μm). (M) Fe^2+^ content in the liver. (N, O) Serum ALT and AST levels (*n* = 8). (P) H&E staining (scale bar = 50 μm). Data are presented as the mean ± SD. *p < 0.05, **p < 0.01, ***p < 0.001, ****p < 0.0001.

To further validate the role of the HSF1/HSPB1 pathway in ferroptosis in the liver of TX mice, we treated with the pathway inhibitor NXP800. The immunohistochemical staining revealed an increase in the expression of HSF1 and HSPB1 in the GDL group compared to the model group. However, the expression of HSF1 and HSPB1 was reduced by NXP800 (Figure 5A–C). Furthermore, in comparison to the GDL group, NXP800 significantly abolished the protein and mRNA levels of HSF1, HSPB1, and FTH1, while it elevated the protein and mRNA levels of TFR1 (Figure 5D–F). Meanwhile, GDL caused a down‐regulation of MDA, ROS, 4‐HNE, Fe^2+^, AST and ALT levels, as well as an increase in SOD and GSH‐Px levels. This treatment also exhibited a beneficial effect in reducing the severity of hepatic pathological injury. However, this trend was reversed by NXP800 (Figure 5G–P). Based on the above results, it is reasonable to infer that the HSF1/HSPB1 pathway inhibitor NXP800 significantly attenuated the protective effects of GDL on ferroptosis in the liver of TX mice.

## 4 DISCUSSION

Liver injury is a symptom experienced by almost every patient with WD, and if timely and effective intervention is not provided, it may lead to fulminant liver failure or even death. Copper chelators and zinc salts are commonly used therapeutic drugs in clinical practice. They have the ability to control the symptoms of the disease but are associated with various adverse reactions, including gastrointestinal dysfunction, allergic reactions, myasthenia gravis and autoimmune diseases.^32^, ^33^ In recent years, TCM has proven to be a viable therapeutic option for managing this condition due to its characteristics of multiple components, multiple targets and multiple pathways, as well as low toxicity and fewer side effects.^34^, ^35^

Ferroptosis is a recently identified type of iron‐dependent programmed cell death that is linked to the evolution of WD liver injury,^5^ but the pathogenic relationship and mechanism between the two have not yet been fully uncovered. Finding effective interventions of TCM to resist ferroptosis and elucidating its mechanisms emerge as a potential avenue for treating WD liver injury. Here, we report that GDL, an effective Chinese herbal compound preparation, exhibits significant protective effects against liver injury in TX mice. Mechanistically, GDL can alleviate WD liver injury by inhibiting ferroptosis via the HSF1/HSPB1 pathway.

TX mice, with pathological changes such as copper accumulation and liver injury in vivo similar to those observed in WD patients, are considered classic animal models for studying WD.^36^ The hepatoprotective effect of GDL has been demonstrated in our study, which is achieved through the alleviation of liver histopathological injury, improvement of mitochondrial structural disruption, reduction of liver copper content and mitigation of liver oxidative stress. Oxidative stress is a significant contributor to lipid peroxidation, which plays a critical role in the process of ferroptosis.^37^ In TX mice, copper overload is accompanied by iron overload, wherein excessive reactive iron provides electrons to generate ROS through the Fenton reaction, thereby promoting the accumulation of lipid peroxidation product MDA, which ultimately leads to the occurrence of ferroptosis. GSH‐Px and SOD are key antioxidant enzymes that eliminate excess MDA and ROS. We found that GDL treatment significantly restored the levels of GSH‐Px and SOD, along with a reduction in the accumulation of ROS and MDA. Previous studies have demonstrated the powerful free radical scavenging and antioxidant properties of GDL, facilitating a better understanding of the process by which GDL counteracts liver lipid peroxidation.^35^

As a vital regulator of anti‐ferroptosis, GPX4 is directly and positively modulated by glutathione (GSH) and protects liver from lipid peroxidation by limiting the level of ROS, thus inhibiting ferroptosis.^38^ Moreover, copper overload can directly induce the aggregation of HSF1 in the cytoplasm, which has been demonstrated to increase mitochondrial oxidative damage, thereby leading to ferroptosis.^39^, ^40^, ^41^ The protein and mRNA levels of ferroptosis markers, GPX4 and HSF1, were found to be substantially elevated following treatment with the ferroptosis inhibitor, Fer‐1. In subsequent studies, it was demonstrated that Fer‐1 exhibits a similar ability to GDL in alleviating liver injury in TX mice, with a significant reduction in Fe^2+^ content and an increase in Fe^3+^ in the liver tissue. These findings provided strong evidence for the involvement of ferroptosis in the development of liver injury in TX mice.

Bioinformatics Analysis and machine learning algorithms provide an opportunity to identify potential diagnostic biomarkers, and therapeutic targets of various diseases.^42^ We found a significantly down‐regulated expression of *Hspb1* in the liver of WD model mice, indicating its potential as a promising target for WD diagnosis and therapy. The cellular cytoskeleton of actin mediated by HSPB1 (an important effector of HSF1) has been demonstrated to play a crucial role in regulating iron homeostasis.^43^, ^44^ Specifically, it functions by inhibiting the iron uptake mediated by TFR1 and upregulating the expression of FTH1. The process inhibits endocytosis and the recycling of transferrin. In this study, the changes in proteins and mRNA involved in iron transport in the liver of TX mice were observed, including increases in TFR1, and decreases in HSF1, HSPB1, and FTH1. Supplementation with GDL reversed these effects and significantly alleviated the accumulation of 4‐HNE (a marker of lipid peroxidation and ferroptosis) and Fe^2+^ in the liver.

**FIGURE 6:**
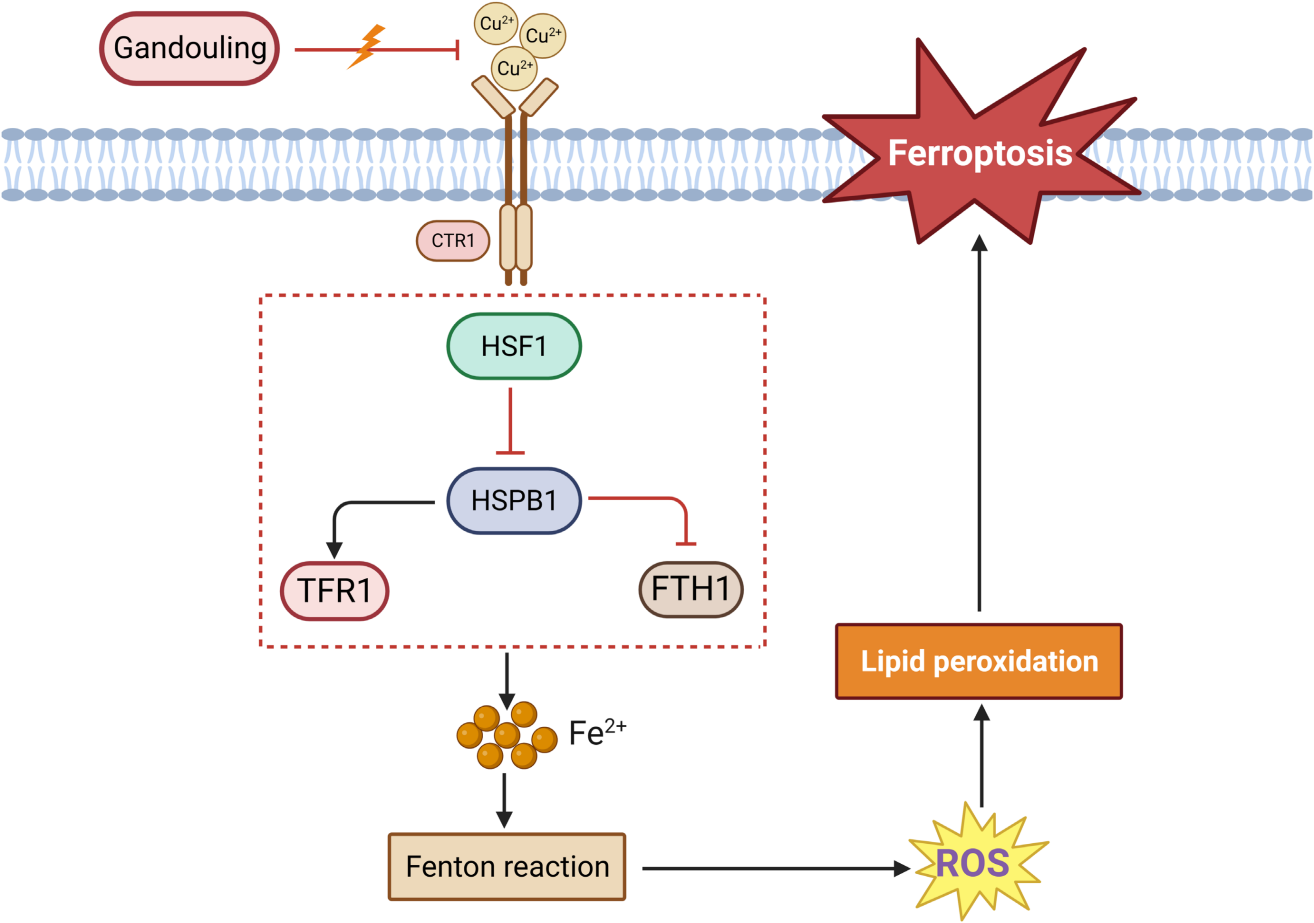
The mechanism of GDL ameliorates WD liver injury through the inhibition of ferroptosis by regulating the HSF1/HSPB1 pathway.

Notably, the involvement of the HSF1/HSPB1 pathway in ferroptosis in cancer has been established.^44^ However, its role in WD liver injury remains unexplored. Hence, we investigated the contribution of the HSF1/HSPB1 pathway to ferroptosis in the liver of TX mice. The results unveiled a decrease in TFR1 expression and an increase in HSF1, HSPB1 and FTH1 expression following treatment with the pathway inhibitor, NXP800. It is of utmost importance that the activation of excessive iron‐induced ROS and subsequent lipid peroxidation, which are triggered by the Fenton reaction, is initiated through this process, consequently inducing ferroptosis. Interestingly, the negative impact of NXP800 was counteracted by GDL treatment. Taken together, iron homeostasis dysregulation and lipid peroxidation in the liver of TX mice can be ameliorated by GDL through the modulation of the HSF1/HSPB1 pathway, thereby inhibiting ferroptosis and alleviating liver injury (Figure 6).

## 5 CONCLUSION

Collectively, these findings suggest that the amelioration of iron homeostasis dysregulation and lipid peroxidation by the HSF1/HSPB1 pathway is critical for preventing ferroptosis in the liver of TX mice, which is also the primary pathway through which GDL exerts its beneficial effects.

## AUTHOR CONTRIBUTIONS

**Chenling Zhao:** Conceptualization (equal); methodology (equal); writing – original draft (equal). **Jie Chen:** Investigation (equal); software (equal). **Liwei Tian:** Investigation (equal); software (equal). **Yuya Wen:** Visualization (equal). **Mingcai Wu:** Writing – review and editing (equal). **Lulu Tang:** Writing – review and editing (equal). **An Zhou:** Methodology (equal). **Wenting Xie:** Supervision (equal). **Ting Dong:** Funding acquisition (equal); supervision (equal).

## FUNDING INFORMATION

This project was supported by grants from the Natural Science Foundation of Anhui Province (2208085MH270, 2108085QH368), the National Training Program for Outstanding Western Medical Professionals in Traditional Chinese Medicine, Second Batch (National Letter of Education for Traditional Chinese Medicine Personnel [2023] No. 90, spyc20230088), the National Natural Science Foundation of China (82205076), and the Key Research and Development Plan Projects of Anhui Province (202204295107020043).

## CONFLICT OF INTEREST STATEMENT

The authors declare that they have no known competing financial interests or personal relationships that could have appeared to influence the work reported in this paper.

## CONSENT FOR PUBLICATION

This manuscript was published with the approval of all authors, and there was no conflict of interest in the submission.

## Data Availability

The data that support the findings of this study are available from the corresponding author upon reasonable request.
